# Functional dichotomy in the 16S rRNA (m^1^A1408) methyltransferase family and control of catalytic activity via a novel tryptophan mediated loop reorganization

**DOI:** 10.1093/nar/gkv1306

**Published:** 2015-11-24

**Authors:** Marta A. Witek, Graeme L. Conn

**Affiliations:** Department of Biochemistry, Emory University School of Medicine, Atlanta GA 30322, USA

## Abstract

Methylation of the bacterial small ribosomal subunit (16S) rRNA on the N1 position of A1408 confers exceptionally high-level resistance to a broad spectrum of aminoglycoside antibiotics. Here, we present a detailed structural and functional analysis of the *Catenulisporales acidiphilia* 16S rRNA (m^1^A1408) methyltransferase (‘*Cac*Kam’). The *apo Cac*Kam structure closely resembles other m^1^A1408 methyltransferases within its conserved SAM-binding fold but the region linking core β strands 6 and 7 (the ‘β6/7 linker’) has a unique, extended structure that partially occludes the putative 16S rRNA binding surface, and sequesters the conserved and functionally critical W203 outside of the *Cac*Kam active site. Substitution of conserved residues in the SAM binding pocket reveals a functional dichotomy in the 16S rRNA (m^1^A1408) methyltransferase family, with two apparently distinct molecular mechanisms coupling cosubstrate/ substrate binding to catalytic activity. Our results additionally suggest that *Cac*Kam exploits the W203-mediated remodeling of the β6/7 linker as a novel mechanism to control 30S substrate recognition and enzymatic turnover.

## INTRODUCTION

Aminoglycosides are potent antimicrobial agents that alter bacterial protein synthesis by inducing defects in the process of decoding by the ribosome (1–6). However, exceptionally high-level aminoglycoside-resistance is achieved by the action of intrinsic and acquired 16S ribosomal RNA (rRNA) methyltransferase enzymes in aminoglycoside-producing and human pathogenic bacteria, respectively (7). These resistance determinants block drug binding by catalyzing the transfer of a methyl group from their cosubstrate *S*-adenosyl-l-methionine (SAM) to the base of a defined target RNA nucleotide within the ribosomal A-site.

Two distinct families of aminoglycoside-resistance 16S rRNA methyltransferases are defined by their methylation target, producing either N7-methyl (m^7^)G1405 or N1-methyl (m^1^)A1408 modifications (8–10). These methylations result in overlapping but distinct aminoglycoside resistance profiles. m^7^G1405 confers resistance exclusively to 4,6-disubstituted 2-deoxystreptamine (4,6-DOS) aminoglycosides, including kanamycin and gentamicin, but not to members of other aminoglycoside structural classes (9,10). In contrast, m^1^A1408 confers resistance to specific aminoglycosides within each structural class, including kanamycin (but not gentamicin) of the 4,6-DOS group, the 4,5-DOS neomycin, and apramycin (9–11). Collectively, these two 16S rRNA modifications confer exceptionally high-level resistance to almost all clinically-relevant aminoglycosides, including the latest generation drugs (7,12).

**Figure 1.**
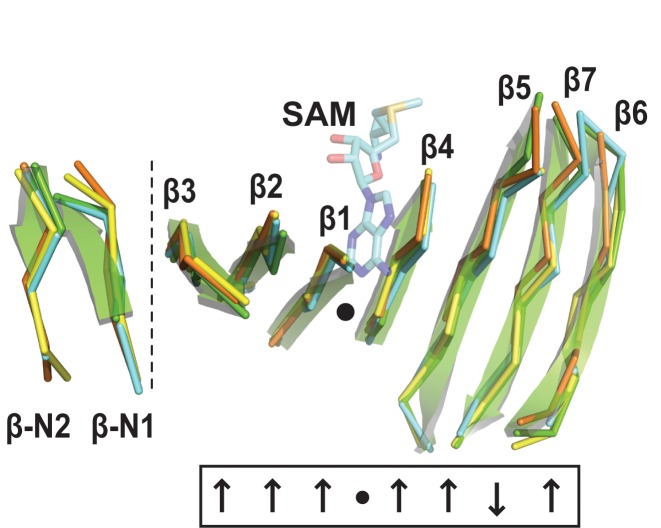
The conserved β-sheet core of the 16S rRNA (m^1^A1408) methyltransferase family. Class I methyltransferases possess a structurally conserved, seven-stranded β-sheet (β1 to β7) with a central topological switch point (black dot) that creates the SAM-binding pocket. The conserved β-sheet core of the 16S rRNA (m^1^A1408) methyltransferases is extended by a short N-terminal extension (β-N1 and β-N2, separated by the vertical dotted line). Structures shown are NpmA (cyan; PDB code 3MTE), KamB (orange; 3MQ2), Kmr (yellow; 4RWZ) and *Cac*Kam from *C. acidiphilia* (green cartoon; this study). The bound SAM (semi-transparent cyan sticks) is from the crystal structure of the NpmA–SAM complex (3MTE).

Three high-resolution structures of the eleven unique members of the 16S rRNA (m^1^A1408) methyltransferase family have been determined to date, including NpmA from an *E. coli* strain ARS3 clinical isolate, KamB from the tobramycin producer *Streptoalloteichus tenebrarius*, and Kmr from *Sorangium cellulosum* So ce56 (13–15). All three possess the conserved Class I methyltransferase SAM-binding fold (16,17), composed of a seven-stranded β-sheet core sandwiched by α-helices. SAM cosubstrate is bound in a cleft formed by a central topological switch point in the β sheet (Figure 1). The 16S rRNA (m^1^A1408) methyltransferases also have expanded sequences at their N-terminus, and between β strands 5 and 6, and β strands 6 and 7 (β5/6 and β6/7 ‘linkers’, respectively) which augment the core SAM-binding fold.

The aminoglycoside-resistance 16S rRNA methyltransferases modify their target nucleotide only in the context of the intact 30S subunit. The molecular basis for this strict substrate requirement was recently revealed for one m^1^A1408 family member, NpmA, for which a structure was determined in complex with 30S and the SAM analog sinefungin (18). NpmA interacts exclusively with the 16S rRNA, making direct contacts via the family-specific augmented β5/6 and β6/7 linkers, and a third region between β strands 2 and 3 (β2/3 linker). The NpmA β2/3 linker forms a rigid, positively charged surface, that docks on a complementary 16S rRNA tertiary surface comprising four 16S rRNA helices. The β6/7 linker plays two critical roles in positioning of the target nucleotide: Arg residue (R207) contacts the A1408 phosphate group to stabilize the ‘flipped’ nucleotide conformation, while the conserved W197 positions the base in the NpmA active site. Finally, the β5/6 linker undergoes a significant conformational change upon 30S binding which positions a Glu residue (E146) to support the functionally critical R207, in addition to making a direct contact to 16S rRNA via R153.

Conservation of the positively charged β2/3 linker among NpmA, KamB and Kmr suggests a common mechanism of initial docking on the same 16S rRNA tertiary surface by the m^1^A1408 methyltransferase family. However, structural and functional studies of each individual enzyme have also pointed to potential differences in the molecular details of β5/6 and β6/7 linker participation in 30S substrate recognition and modification. The β5/6 linker appears dynamic and adopts a different conformation in each of the three enzymes (13–15). Assignment of equivalent functional roles in 30S binding and A1408 base flipping to specific β5/6 linker residues in KamB or Kmr is challenging because of the low sequence identity. Second, whereas KamB and NpmA share a defined β6/7 linker structure that caps the SAM-binding pocket, the β6/7 linker is disordered in Kmr (15). Remarkably, Kmr does not bind SAM, suggesting a unique mechanism of controlling 30S recognition and A1408 modification where SAM cosubstrate binding is facilitated by 30S (15). In NpmA, a single β6/7 linker residue, R207, is critical for A1408 base flipping (18). In KamB this function is predicted to be accomplished in concert by two other β6/7 linker Arg residues whose substitution ablates activity *in vivo* (13). In contrast to both these examples, no single substitution of a β6/7 linker basic residue was found to reduce Kmr activity (15).

These studies also revealed unexpected differences in the apparent contribution of several conserved residues to the activity of NpmA, KamB and Kmr. For example, D55A, E88A or S195A substitution in the SAM-binding pocket had no effect on NpmA activity *in vivo* despite eliminating SAM binding *in vitro*, whereas equivalent substitutions in KamB reduced or completely abrogated activity *in vivo* (13,14). In further contrast, Kmr activity *in vivo* was found to be generally resistant to the effects of single amino acid substitutions in the SAM-binding pocket (15). The absence of functional equivalence at these conserved residues suggests significant flexibility in the SAM-binding pocket among the members of 16S rRNA (m^1^A1408) methyltransferase family.

In summary, among the 16S rRNA (m^1^A1408) methyltransferases studied to date, substantial evidence points to the potential for varied molecular mechanisms underpinning 30S recognition, base flipping and activation of catalysis. Such properties are important to fully understand as they have the potential to confound efforts to develop inhibitors of these resistance determinants. Therefore, as a basis for developing broader insight into this important resistance enzyme family, we previously reported the cloning and initial functional analysis of four previously uncharacterized 16S rRNA (m^1^A1408) methyltransferases (19). Here, we describe detailed structural and functional analyses of one of these enzymes, the kanamycin-resistance methyltransferase from *Catenulisporales acidiphilia* (‘*Cac*Kam’). Our findings support a mechanistic dichotomy within the 16S rRNA (m^1^A1408) methyltransferase family but also suggest that *Cac*Kam uses structural remodeling of the functionally critical β6/7 linker as a novel mechanism to control its m^1^A1408 modification activity.

## MATERIALS AND METHODS

### Protein expression, purification and mutagenesis

We previously reported the cloning, expression and purification of the 16S rRNA (m^1^A1408) methyltransferase *Cac*Kam from *C. acidiphilia* with an N-terminal hexahistidine tag (19). Briefly, for the studies reported here, *Cac*Kam was expressed in *E. coli* BL21 (DE3) cells grown in lysogeny broth (LB) at 37°C with induction of protein expression by addition of isopropyl β-d-1-thiogalactopyranoside (IPTG) to 0.5 mM final concentration. Cells were lysed by sonication and *Cac*Kam purified by consecutive Ni^2+^-affinity and gel filtration chromatographies.

Site-directed mutagenesis was performed using a two-step protocol involving an initial PCR with standard DNA primers (19–27 nts), one containing the desired mutation, to generate ∼60–630 base pair ‘megaprimers’ for use in a second round of whole plasmid amplification (20). All variant *Cac*Kam and KamB proteins were expressed and purified as for wild-type *Cac*Kam.

### Protein crystallization, X-ray data collection and structure determination

Crystallization of wild-type *apo Cac*Kam (250 μM) was carried out at 20°C by hanging-drop vapour diffusion with a final optimized crystallization solution consisting of 0.03 M Bis-Tris (pH 5.5) and 25.5% polyethylene glycol (PEG) 3350. Crystals formed after ∼7 days and were cryoprotected by addition of glycerol (20% final concentration) prior to harvesting and flash freezing in liquid nitrogen. To obtain a complex of wild-type *Cac*Kam with S-adenosylhomocysteine (SAH), preformed *apo Cac*Kam crystals were soaked in crystallization solution containing 20 mM SAH for ∼8 h before cryoprotection and harvesting. *Apo Cac*Kam-W203A (480 μM) was crystallized in 1.85 M ammonium sulfate, 0.08 M Tris, pH 8.5, and 3.5% dimethyl sulfoxide (DMSO). The structure of a *Cac*Kam-W203A complex with SAM was obtained by soaking preformed crystals with crystallization solution containing 10 mM SAM. *Cac*Kam-D21A (400 μM) was crystallized in 0.2 M ammonium acetate, 0.1 M sodium acetate trihydrate, pH 4.6 and 30% PEG 4000. All crystals were cryoprotected and harvested as described for the wild-type protein.

X-ray diffraction data were collected at the Southeast Regional Collaborative Access Team (SER-CAT) 22-ID beamline at the Advanced Photon Source, Argonne National Laboratory. Indexing, integration of diffraction images, and scaling of the diffraction data were carried out in HKL2000 (21). The structure of wild-type *apo Cac*Kam was determined by molecular replacement using KamB (PDB code: 3MQ2, chain A) as a search model in PHENIX AutoMR/ Phaser (22). The refined wild-type *apo Cac*Kam structure was subsequently used as the molecular replacement starting model for all additional *Cac*Kam structures. In each case, the identified solution was used as the input for automatic model building in PHENIX AutoBuild (22). Following manual model adjustment in *Coot* (23), the complete models were subjected to rigid-body TLS parametrization via the TLSMD server (24) followed by refinement with PHENIX (22). The quality of each model was assessed with MolProbity (25) and PDB_REDO (26). Final refinement of the *Cac*Kam-203A:SAM structure was performed with REFMAC (27) using optimized refinement settings output by PDB_REDO. All structure factors and refined coordinates have been deposited in the Protein Data Bank (PDB). Final X-ray diffraction data collection and refinement statistics are presented in Supplementary Table S1. Molecular and electron density illustrations were prepared with PyMOL (28).

### Limited proteolysis with trypsin

Proteins (30 μM) were subjected to limited proteolysis with trypsin (0.05 to 0.1 μg/μl final concentration) for 5 min at room temperature in 10 mM HEPES pH 7.5 and 500 mM NaCl. The reaction was terminated by boiling samples for 5 min in SDS-PAGE gel loading dye. Reaction products were resolved on a 16% SDS-PAGE gel and the fragment masses estimated using AlphaView (Protein Simple; Santa Clara, CA, USA) from their migration relative to full-length *Cac*Kam and protein molecular weight standards. Trypsin cleavage sites were deduced by consideration of these masses and the solvent accessibility of each potential site in the structure determined using the POPS solvent accessible surface area calculations server (29). The identity of the smallest digestion product was confirmed by liquid chromatography coupled to tandem mass spectrometry (LC-MS/MS) following in-gel digestion with trypsin using an Orbitrap Fusion mass spectrometer (Thermo Scientific) at the Emory Integrated Proteomics Core.

### Antibiotic minimum inhibitory concentration (MIC) assays

Kanamycin MIC assays were performed in a 96-well plate format as described previously (19). In each well, 100 μl LB supplemented with IPTG (5 μM) and kanamycin (0–2048 μg/ml) was inoculated with a further 100 μl LB containing 1 × 10^5^ cfu/ml *E. coli* BL21 (DE3) cells harboring either empty (control) or a *Cac*Kam-encoding plasmid. Plates were incubated with shaking for 24 h at 37°C after which the absorbance at 600 nm for each well was recorded. The MIC was defined as the lowest concentration of antibiotic that inhibited growth (OD_600_ < 0.05 above background).

### Isothermal titration calorimetry (ITC)

ITC measurements were performed at 25°C using an Auto-iTC_200_ microcalorimeter (Malvern/ MicroCal). Titration experiments comprised 16 × 2.5 μl injections of SAM (1.5–3.0 mM) or SAH (0.5–1.3 mM) into the sample cell containing wild-type or variant *Cac*Kam or KamB protein (50–80 μM). The titration isotherm was fit using the single binding site model implemented in Origin 7 software in order to determine the binding affinity (*K*_d_) for each protein-ligand pair.

### 30S subunit *in vitro* methylation assays

30S substrate methylation was determined using a reverse transcription (RT) assay with *E. coli* MRE600 30S subunits purified as described previously (30). Various concentrations of methyltransferase protein (10–1000 pM) were incubated with a fixed amount of 30S (100 pM) for 1 hour at 37°C in 30S methylation assay buffer (10 mM HEPES–KOH (pH 7.5), 10 mM MgCl_2_, 50 mM NH_4_Cl, and 5 mM β-mercaptoethanol) containing SAM (1 mM). The reaction was terminated by phenol/chloroform extraction followed by ethanol precipitation to recover 16S rRNA. The reaction product was analyzed by RT using a ^32^P-labeled DNA primer complementary to *E. coli* 16S rRNA nucleotides 1457–1473. Extension products were run on 10% PAGE-urea gels and visualized using a Typhoon Trio phosphorimaging system (GE Healthcare). Methylation of A1408 at the N1 position (m^1^A1408) produces a strong stop in the RT reaction resulting in an intense band on the gel at the position corresponding to nucleotide C1409. Band intensities at different protein concentrations were determined using ImageQuant TL Software (GE Healthcare) and these values converted to fraction methylated, assuming complete methylation occurs in the reaction with 10-fold excess enzyme.

## RESULTS AND DISCUSSION

### The *Cac*Kam β6/7 linker adopts a novel extended conformation

The *apo* form of the 16S rRNA (m^1^A1408) methyltransferase *Cac*Kam crystallized in space group *P*2_1_2_1_2_1_, and its structure was determined by molecular replacement and refined to 1.7 Å-resolution. Our recombinant *Cac*Kam consists of an N-terminal hexahistidine tag linked to the 250 residue methyltransferase by a thrombin cleavage site (19). A single polypeptide chain was identified in the asymmetric unit for which clear density allowed unambiguous modeling of *Cac*Kam residues 8–226. The locations of the N-terminal tag (residues −16 to −1), and the *Cac*Kam N- and C-termini (residues 1–7 and 227–250, respectively) could not be determined.

**Figure 2.**
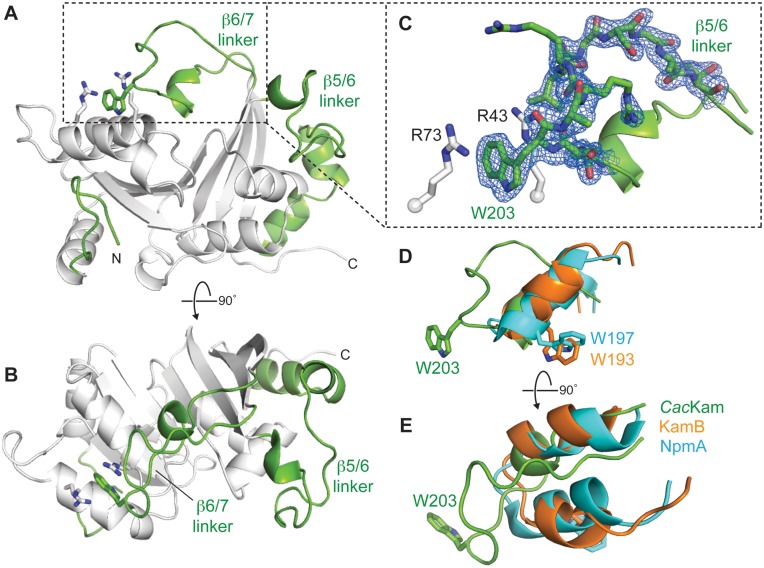
The crystal structure of *apo Cac*Kam methyltransferase reveals a novel conformation of the functionally critical β6/7 linker. (**A** and **B**) Two orthogonal views of the 1.7-Å resolution crystal structure of *apo Cac*Kam highlighting (green) the three regions which augment the Class I methyltransferase fold at the N-terminus (N), and between β-strands 5 and 6 (β5/6 linker), and 6 and 7 (β6/7 linker). Interacting residues stabilizing the novel β6/7 linker conformation, R43/ R73 (white sticks) and W203 (green sticks), are also shown. (**C**) Zoomed in view of the *Cac*Kam β6/7 linker and the cation-π-cation interaction of residues R73/W203/R43. Residues S201-T212 of the β6/7 linker are shown in 2*F*_o_ − *F*_c_ electron density contoured at 1.0*σ*. (**D** and **E**) Two orthogonal views comparing the β6/7 linker structure of *Cac*Kam (green), NpmA (cyan) and KamB (orange), shown in the same orientation as the view of panel A. Functionally critical NpmA (W197) and KamB (W193) tryptophan residues equivalent to *Cac*Kam W203 are also shown.

*Cac*Kam has a SAM-binding core fold comprised of a seven-stranded β-sheet with a central topological switch point, characteristic of Class I methyltransferases (16) (Figures 1 and 2A). Additionally, in common with structures of other 16S rRNA (m^1^A1408) methyltransferases, the core fold of *Cac*Kam is augmented by a short N-terminal β-hairpin and expanded β5/6 and β6/7 linker regions (Figure 2A). In contrast to other family members, however, the *Cac*Kam β6/7 linker (residues V191-V215) forms an extended loop that makes unexpected interactions with an adjacent surface via the conserved W203. This β6/7 linker structure is surprising as both this linker and the partially occluded adjacent surface containing the β2/3 linker are critical for the activity of other 16S rRNA (m^1^A1408) methyltransferases. The β2/3 linker is rich in conserved basic residues and docks against a complementary, conserved 16S rRNA tertiary surface in the 30S-NpmA complex (18). Additionally, in both NpmA and KamB, the β6/7 linker forms a similar, more compact structure (Figure 2D and E) that caps SAM-binding pocket and, in the 30S-NpmA complex, orients the residue equivalent to *Cac*Kam W203 (NpmA W197) into the enzyme active site where it positions the flipped A1408 base for modification (13,18). Given these critical roles for both the β6/7 linker and the adjacent occluded surface in other 16S rRNA (m^1^A1408) methyltransferases, we set out to determine whether the unexpected *Cac*Kam β6/7 linker conformation might play a specific functional role in *Cac*Kam activity.

We first asked whether the *Cac*Kam β6/7 linker structure might simply reflect a stable conformation of a dynamic loop in the context of packing within the crystal. The extented β6/7 linker structure is potentially stabilized by both intramolecular and intermolecular (crystal packing) interactions. Residue W203 is held in its extended conformation by a double cation-π stacking interaction with two arginine residues within the same molecule, R43 and R73 of the β1/2 and β2/3 linkers, respectively, while the adjacent R206 makes a potentially important crystal packing contact via a salt bridge with D21 of a symmetry related molecule (Figure 2A and B, and Supplementary Figure S1A). To assess the relative contributions of these two interactions to the observed β6/7 linker structure, individual D21A and W203A substitutions were created for further functional and structural analyses. *Cac*Kam-D21A had an activity equivalent to the wild-type enzyme as determined by the resistance conferred in *E. coli* to kanamycin (MIC >1024 μg/ml). In contrast, substitution of the universally conserved W203 to alanine resulted in complete loss of resistance (Table 1), consistent with previous analyses of equivalent substitutions in KamB and NpmA (13,14). This observation clearly points to a critical functional role for *Cac*Kam W203 despite its unusual location in our structure of the *apo* enzyme.

**Table 1.** Impact on *Cac*Kam activity and ligand interactions of residue substitutions around the SAM-binding pocket

Amino acid substitution	Kanamycin MIC (μg/ml)	Ligand binding^a,b^	
		SAM *K*_d_ (μM)	SAH *K*_d_ (μM)
-	>1024	44	2.7
D36A	8	*NB*	*NB*
D61A	8	*NB*	*NB*
R66A	512	21	2.9
E94A	16	29	2.2
K115A	256	48	5.0
S201A	16	110	1.6
S202A	8	24	2.9
R43A/R73A	16	35	2.4
W113A	16	31	1.9
W113F	16	103	11
W203A	16	68	5.1
W203F	16	24	1.9

^a^Data for wild-type *Cac*Kam are from reference (19).

^b^NB, denotes no binding detected.

Crystal structures were next determined for each *Cac*Kam variant to assess the effects of the D21A and W203A substitutions on β6/7 linker structure and crystal packing interaction. Despite the loss of a potentially important crystal packing contact, *Cac*Kam-D21A crystallized in the same space group as the wild-type enzyme with essentially identical unit cell parameters and crystal packing arrangement. Critically, the *Cac*Kam-D21A β6/7 linker adopts an identical extended conformation (Supplementary Figure S2) retaining the double cation-π stacking interaction of W203 with R43 and R73. In contrast, although *Cac*Kam-W203A also crystallized in space group *P*2_1_2_1_2_1_, the substitution results in a new unit cell with an altered packing arrangement and two molecules in the asymmetric unit (Supplementary Figure S1B). Changes in the *Cac*Kam-W203A structure are confined to the β6/7 linker, which is disordered in both chains (Supplementary Figure S2). Additionally, D21 is no longer involved in crystal packing interactions made by either *Cac*Kam-W203A molecule. Thus, only direct disruption of the interaction of W203 with R43/ R73 through the W203A substitution is sufficient to induce changes in the β6/7 linker structure and the crystal lattice. Together, these structures indicate that the R43/W203/R73 interaction is the major driver of the novel β6/7 linker structure observed in the crystal.

To further assess the contribution of W203 to the *Cac*Kam β6/7 linker conformation in solution, we probed the structure of wild-type and variant *Cac*Kam proteins by limited trypsin proteolysis. SDS-PAGE analysis of trypsin treated wild-type *Cac*Kam revealed at least four distinct fragments (bands b–e, Supplementary Figure S3) in addition to the full-length protein (28.6 kDa; band a). Analysis of fragment migration using AlphaView yielded estimated masses of 27.3, 24.0, 23.2, and 22.0 kDa for bands b–e, respectively. Of all potential trypsin cleavage sites, surface accessibility, location within the *Cac*Kam structure and resulting fragment sizes suggested residues R-3, K14, R206 and R226 as the most likely sites of cleavage contributing to these stable fragments (Supplementary Figure S3). The *Cac*Kam-R206A variant eliminated two bands (c and d), confirming these fragments are generated by direct cleavage of the β6/7 linker, most likely via R206 single (band c) and R-3/R206 double cleavage (band d). In contrast, the trypsin cleavage patterns (though not band intensities) of W203A, W203F and R43A/R73A substituted proteins were the same as the wild-type protein. The other fragments therefore must be generated by trypsin cleavages outside the β6/7 linker, and likely at R-3 or R226 (band b), and K14/ R226 double cleavage (band e). Consistent with the latter assignment, peptides corresponding to the N- and C-terminal sequences were absent in mass spectrometry analysis of band e. Critically, in terms of β6/7 linker structure and dynamics in solution, the intensity of cleavage at R206 (bands c and d) is increased upon W203A, W203F or R43A/R73A substitution, consistent with increased flexibility in the absence of the R43/W203/R73 double cation–π interaction to stabilize the β6/7 linker conformation. Thus, these data additionally support the *Cac*Kam β6/7 linker adopting an extended structure in solution as observed in the crystal structure.

The unusual conformation of the *Cac*Kam β6/7 linker and its partial occlusion of the adjacent surface containing residues R43 and R73 are incompatible with the known, critical roles played by these regions in SAM binding, 30S substrate recognition and A1408 modification by other 16S rRNA (m^1^A1408) methyltransferases (13,14,18). We therefore speculated that SAM binding, 30S binding, or potentially a combination of both, might drive functional remodeling of the β6/7 linker to allow this region to contribute to enzyme activity in the same manner as for other family members; alternatively, *Cac*Kam could use a unique molecular mechanism to achieve substrate recognition and m^1^A1408 modification.

### Binding of SAM or SAH may prime the β6/7 linker for reorganization

To assess whether cosubstrate binding might influence the conformation of the β6/7 linker we attempted to co-crystallize wild-type *Cac*Kam with either SAM or SAH. Despite *Cac*Kam having comparable ligand binding affinities to other 16S rRNA (m^1^A1408) methyltransferases (14,19), these efforts were unsuccessful. However, a 2.7 Å resolution structure of the *Cac*Kam-SAH complex was obtained by soaking preformed crystals *of apo Cac*Kam with SAH. This complex structure revealed that the SAM-binding pocket is largely preformed in the *apo* protein (Supplementary Figure S4). While the β6/7 linker does not undergo a complete reorganization to a structure resembling that of KamB or NpmA which caps the SAM-binding pocket, SAH binding nonetheless appears to induce greater β6/7 linker flexibility reflected by significantly higher *B*-factors relative to the remainder of the protein, which is essentially unchanged (Supplementary Figure S2).

We next exploited the altered crystal packing and constraints on the β6/7 linker in the *Cac*Kam-W203A crystal form to obtain a complex structure by soaking preformed crystals with SAM. In contrast to the *apo* structure of *Cac*Kam-W203A, SAM binding allowed the peptide backbone path of the β6/7 linker of one molecule (chain B) to be reliably modeled, albeit with significantly higher *B-*factors, in a conformation that is partially refolded on the SAM-binding pocket (Supplementary Figures S1C and S2). The β6/7 linker of the second molecule in the asymmetric unit (chain A), however, remained disordered despite the presence of SAM in the ligand binding pocket. Interestingly, while the fully disordered linker of chain A is completely unrestrained by crystal packing, the β6/7 linker of chain B is adjacent to a symmetry related molecule. This molecular crowding in the crystal potentially mimics interaction with 30S and contributes to the observed closure of the linker exclusive to chain B.

We conclude from these additional structures that cosubstrate binding alone likely does not drive complete reorganization of the β6/7 linker to a structure resembling that of NpmA or KamB. Instead this process must require direct interaction with the 30S substrate. However, significantly increased β6/7 linker *B*-factors in the *Cac*Kam-SAH complex, and the conversion of one *Cac*Kam-W203A β6/7 linker from fully disordered to partially closed in the *apo* form and SAM-bound forms, respectively, are both consistent with SAM binding being a prerequisite for β6/7 linker remodeling induced by interaction with 30S substrate.

### Functional analyses of the SAM-binding pocket reveal differences in dependency on conserved residues among 16S rRNA (m^1^A1408) methyltransferases

**Figure 3.**
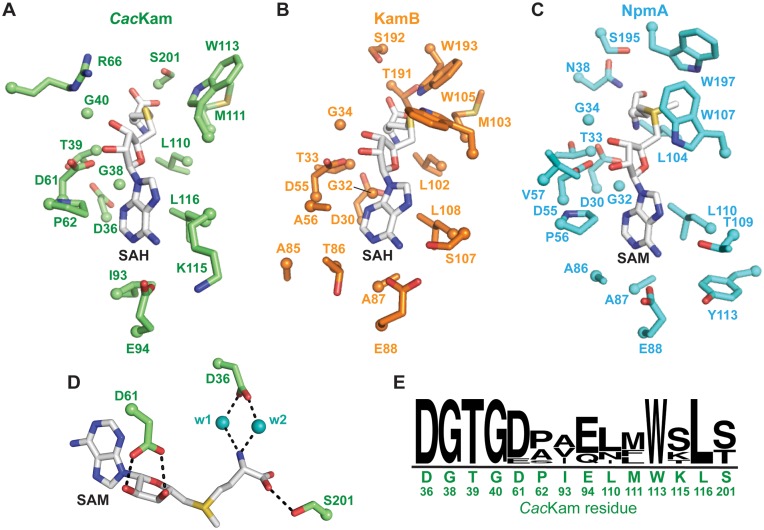
*Cac*Kam possesses a conserved SAM-binding pocket. Comparison of residues forming the cosubstrate binding pocket in crystal structures of (**A**) *Cac*Kam-SAH, (**B**) KamB-SAH and (**C**) NpmA-SAM complexes. (**D**) View of the conserved and functionally essential water-mediated interactions in the SAM-binding pocket, shown for the *Cac*Kam-W203A complex with SAM. (**E**) WebLogo (41) plot of amino acid conservation in the SAM-binding pocket from ClustalW (42) alignment of unique 16S rRNA (m^1^A1408) methyltransferase sequences. The *Cac*Kam residue type and number is shown under the plot.

The finding that SAM binding may be necessary to prime the β6/7 linker for reorganization lead us to ask whether molecular details of the *Cac*Kam interaction with SAM could be identified that might underpin such a connection between cosubstrate/ 30S substrate binding and activation of methyltransfer activity. Inspection of the SAM-binding pocket reveals that the cosubstrate orientation and interactions with *Cac*Kam are essentially identical to those of the KamB-SAH (PDB code 3MQ2) and NpmA-SAM (PDB code: 3MTE) complexes. Conserved hydrophobic and hydrogen bonding interactions are made with the adenine base, ribose and homocysteine moiety of SAH (Figure 3A–C). Binding of SAH to wild-type *Cac*Kam is accommodated by a small (∼1.5 Å) repositioning of the protein backbone encompassing the conserved GxGx(G) motif (^38^GTGDA^42^ in *Cac*Kam). The G38 backbone carbonyl forms a hydrogen bond with the SAH amino group, which is additionally positioned by two conserved residues, via hydrogen bonding interaction with the L110 backbone carbonyl group and a water-mediated interaction with the side chain of D36 (Figure 3A and D). As the β6/7 linker remains in an extended conformation in the *Cac*Kam-SAH structure, interaction with the SAH carboxyl group is limited to a single hydrogen bonding interaction with S201. The hydroxyl groups of the SAH ribose moiety form two hydrogen bonds with the highly conserved aspartic acid residue, D61, while the adenine base is bound in a hydrophobic pocket formed by residues L116, I93 and P62, and additionally positioned by a hydrogen bond between the adenine N6 and the side chain of *Cac*Kam residue E94 (Figure 3A).

To assess the contribution of the interactions observed in the structure between *Cac*Kam and SAH to enzyme-cosubstrate affinity and activity, individual Ala substitutions were made at each residue involved in a side-chain mediated hydrogen bonding interaction. Enzyme activities and ligand binding properties of each Ala-substituted *Cac*Kam protein were assessed by measurements of kanamycin MIC and ITC analyses of SAM or SAH binding affinity, respectively. We found that R66A and K115A substitutions modestly impacted activity while the D36A, D61A and E94A variants were completely unable to confer resistance (Table 1). Further, no SAM or SAH binding could be detected for either D36A or D61A (Table 1). These data for D36A and D61A are consistent with previous analyses of KamB and NpmA which showed that single Ala substitution of D30 and D55 in KamB and NpmA, respectively also abrogated activity of the enzyme. In the case of Kmr, while D30A abrogated activity, D55A substitution had a more modest impact on the kanamycin MIC conferred (15). These results thus further confirm the essential nature of the interactions made by these universally conserved Asp residues for cosubstrate binding and enzyme activity.

In contrast to the impacts of changes at D30 and D55, the effect of the E94A substitution was unexpected. Despite the inability of *Cac*Kam-E94A to confer kanamycin resistance, no effect on SAM or SAH binding affinity was observed (Table 1) as would be expected if E94 directly contributes to cosubstrate binding via its interaction with the SAM adenine N6 (Figure 3A). Curiously, these results also directly contrast the effects of an equivalent change in NpmA which results in a protein that does not bind SAM or SAH *in vitro* but maintains a wild-type ability to confer kanamycin resistance (14). Like *Cac*Kam, the same substitution in KamB (E88A) significantly impacts activity (kanamycin MIC 256 μg/ml) but SAM and SAH binding affinities were not previously determined. We therefore measured the KamB-E88A binding affinity for SAM and SAH by ITC and obtained *K*_d_ values of 56 and 5.3 μM, respectively. These values are identical to (SAM) and modestly weaker (∼5-fold; SAH) than the wild-type enzyme, respectively (15). Thus, the *Cac*Kam-E94A and KamB-E88A substitutions had very similar effects, in direct contrast to the equivalent change in NpmA, where the impact upon enzyme activity was not correlated with loss of SAM-binding affinity.

The β6/7 linker residues S195 and T191 of NpmA and KamB, respectively interact with the carboxyl group of SAM (Figure 3B and C). However, an apparent difference in the contribution of S195/ T191 to SAM binding, and thus NpmA and KamB activity, was previously observed (13,14): a T191A substitution in KamB resulted in an enzyme unable to confer resistance, while the equivalent change in NpmA (S195A) disrupted SAM binding but had no effect on the kanamycin MIC. Ala substitution of the equivalent (S201) and an adjacent (S202) *Cac*Kam residue each resulted in an enzyme unable to confer resistance to kanamycin in the MIC assay (Table 1). However, ligand binding was largely unaffected in both proteins with only the *Cac*Kam-S201A affinity for SAM modestly reduced (2-fold) from the wild-type enzyme (Table 1). As *K*_d_ values for KamB-T191A were not previously determined, we additionally measured SAM and SAH binding and obtained values of 48 and 5.9 μM, respectively; as for KamB-E88A these values are identical to (SAM) and modestly weaker (∼5-fold; SAH) than the wild-type enzyme (15).

Collectively, these results reveal that within the context of an apparently conserved SAM-binding pocket, residues E94 and S201 in *Cac*Kam and their equivalents in KamB make fundamentally different contributions to enzyme activity than for the pathogen-derived enzyme, NpmA. Specifically, while these residues in *Cac*Kam and KamB do not appear to contribute significantly to SAM/SAH affinity in the absence of the 30S substrate, they are nonetheless critical for activity and potentially couple SAM binding with substrate recognition, and activation of methyltransferase activity (Supplementary Table S2). In contrast, in NpmA each residue contributes significantly to SAM binding in isolation, but substitutions of these residues do not result in a deficit in activity. As previously proposed, the effects of these mutations must therefore be overcome by some other aspect of NpmA interaction with its 30S substrate (14,15). Thus, fundamentally different mechanisms of control of modification activity via interactions with SAM and 30S subunit appear to exist among these 16S rRNA (m^1^A1408) methyltransferase family members.

### Conformational remodeling of the β6/7 linker upon 30S recognition is critical for A1408 base flipping

**Figure 4.**
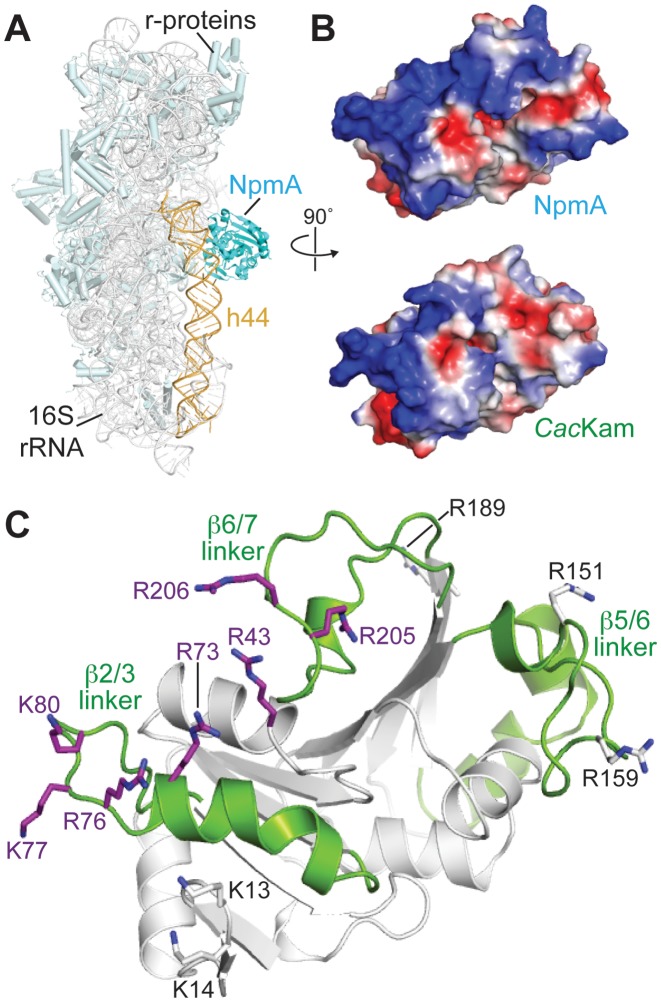
*Cac*Kam residues that mediate interaction with 30S substrate. (**A**) Structure of the 30S-NpmA complex (4OX9) highlighting the enzyme binding site at the top of h44, within the decoding center. (**B**) *Top*, an orthogonal view of NpmA showing the 30S interaction surface in electrostatic potential representation. Regions of the most negative electrostatic potential are in red and most positive are in blue. *Bottom*, equivalent view and electrostatic surface potential of *Cac*Kam, produced by alignment of the *apo Cac*Kam structure with 30S-bound NpmA. (**C**) Cartoon of the *Cac*Kam structure in the same orientation as panel B, highlighting three regions (β2/3, β5/6, and β6/7 linkers) equivalent to those which mediate interaction of NpmA with 30S (18). Residues substituted to alanine (Table 2) are shown as sticks; those resulting in enzymes with reduced (MIC < 256 μg/ml) or comparable (MIC > 512 μg/ml) ability to confer resistance to kanamycin compared to the wild-type enzyme are colored purple and white, respectively.

**Figure 5.**
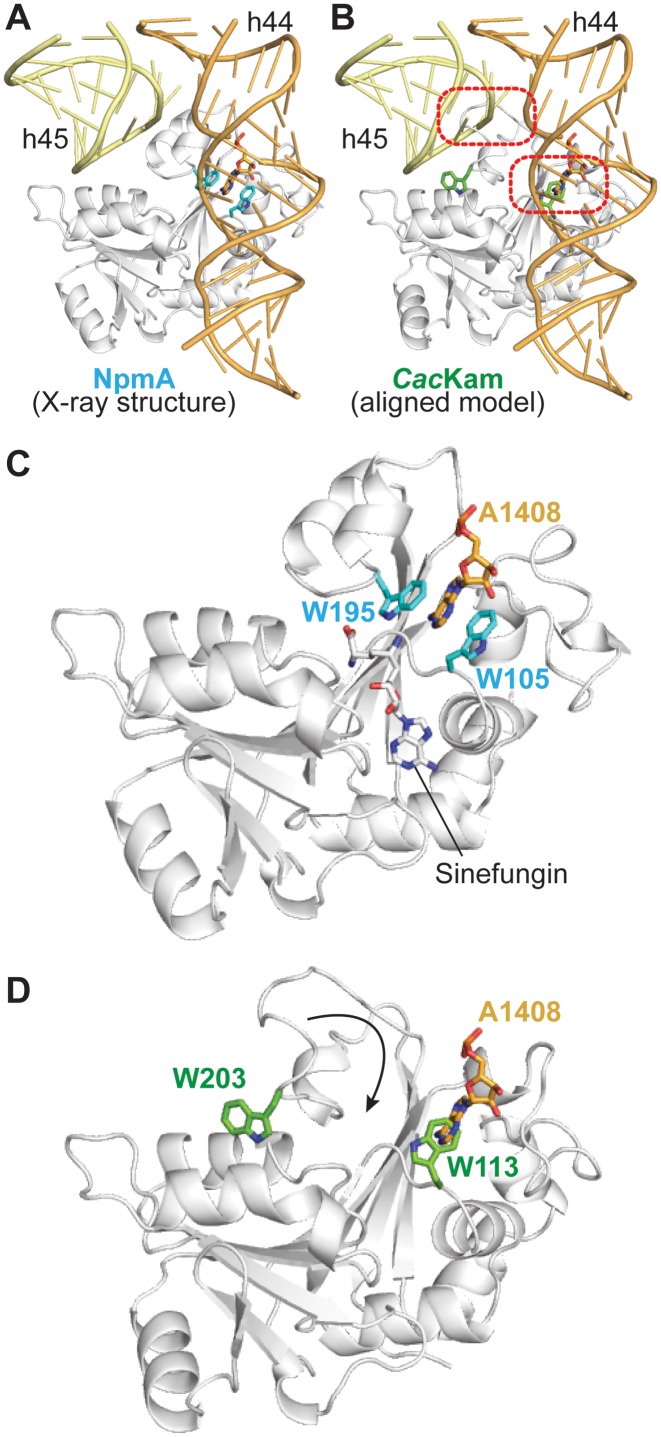
Interaction with 30S requires remodeling of the *Cac*Kam β6/7 linker. (**A**) View of NpmA bound to the 16S rRNA surface formed by h44 and h45 (yellow and orange, respectively). The universally conserved pair of Trp residues and the A1408 target nucleotide are shown as sticks. (**B**) Equivalent view of a model of the *Cac*Kam-30S complex generated by alignment of *Cac*Kam to the 30S-bound NpmA structure. Two sites of clash between *Cac*Kam and 16S rRNA are highlighted (dashed red boxes): the *Cac*Kam β6/7 linker overlaps with h45 of 30S, and a rotation of W113 is also necessary to accommodate the A1408 base in the enzyme active site. Zoomed views showing only (**C**) NpmA and (***D***) *Cac*Kam in the same orientation. A movement of *Cac*Kam W203 of >10 Å is necessary to adopt an equivalent position to NpmA W195 for interaction with A1408.

The NpmA β2/3 and β6/7 linkers form an extended, positively charged surface that interacts with a conserved 16S rRNA tertiary surface in the 30S-NpmA complex (Figure 4A and B) (18). KamB possesses the same positively charged surface and can be docked on the 30S by alignment with 30S-bound NpmA, suggesting that the interaction between these complementary surfaces may be a conserved feature of m^1^A1408 methyltransferase-30S recognition (18). However, although part of this positive surface is present in *Cac*Kam, it is interrupted at the site of the structurally altered β6/7 linker (Figure 4B). As a result, while basic residues present in the equivalent regions of NpmA and KamB are conserved in *Cac*Kam, they are dispersed rather than confined to a single contiguous surface. Additionally, although the β2/3 linker can be positioned similarly to NpmA by alignment of the *apo Cac*Kam structure with 30S-bound NpmA, as expected extensive conformational changes are necessary to relieve clashes between *Cac*Kam residues 202–212 and 16S rRNA h45 in order to accommodate the β6/7 linker in a similar orientation (Figure 5A and B). A reorientation of W113 in the enzyme active site is also necessary to accommodate the flipped A1408 base.

To assess the mechanism of *Cac*Kam recognition of the 16S rRNA tertiary surface, single alanine substitutions of basic residues were introduced, guided by sequence alignment and the structure of *apo Cac*Kam (Figure 4C). Individual substitution of basic residues in each of the β1/2 (R43A), β2/3 (R73A, K77A, and K80A) and β6/7 linkers (R206A) eliminated resistance to kanamycin (Table 2). Additional substitutions in these regions and at the *Cac*Kam N-terminus (K13A and K14A) had more modest effects on activity, while substitution of β5/6 linker basic residues (R151 and R159) resulted in little to no decrease in MIC (Table 2). Together, these results confirm that the same molecular surface with its cluster of conserved basic residues is essential for *Cac*Kam-30S interaction as for other 16S rRNA (m^1^A1408) methyltransferases (Figure 4C).

**Table 2.** Impact on *Cac*Kam activity of substitutions of residues predicted to mediate interaction with 30S

Protein region	Amino acid substitution	Kanamycin MIC (μg/ml)
N-terminus	K13A	512
	K14A	1024
β1/2 linker	R43A	16
β2/3 linker	R73A	16
	R76A	256
	K77A	16
	K80A	16
β5/6 linker	R151A	1024
	R159A	>1024
β6/7 linker	R189A	>1024
	R205A	256
	R206A	16

The dramatic impact on enzyme activity of alanine substitution at R206 and other β6/7 linker residues described above (S201, S202 and W203; Table 1), is consistent with these residues performing critical roles during catalysis that would necessitate β6/7 linker reorganization. First, as noted above we speculate that S201 and/ or S202 (together with E88 at the opposite end of the bound SAM) are responsible for coupling 30S binding and optimal interaction with SAM cosubstrate, and that this role is critical for *Cac*Kam and KamB but not for NpmA. Second, *Cac*Kam R206, either alone or in conjunction with R205, appears the most likely candidate to fulfil the essential role of stabilizing the phosphate backbone conformation of the flipped A1408 nucleotide. NpmA employs a single functionally critical Arg residue (R207) supported by interaction with residue E146 which is repositioned to fulfil its role by the 30S-binding induced β5/6 linker conformational change. In KamB substitutions at two residues, R196A and R201, resulted in ablation of activity suggesting they may act in concert to stabilize the flipped A1408 conformation (13). In contrast, *Cac*Kam has only a single absolutely critical residue (R206) while no equivalent substitution was identified in Kmr (15). Thus, while potentially employing a similar strategy to stabilize the flipped A1408, the molecular details of this important aspect of their action may also differ significantly among the members of the 16S rRNA (m^1^A1408) methyltransferase family (Supplementary Figure S5). Third, as revealed by the NpmA-30S complex structure (18), two universally conserved Trp residues, equivalent to *Cac*Kam W113 and W203, have a joint, critical role in sequestering the flipped A1408 base in the enzyme active site adjacent to the bound SAM (Figure 5C). Although the extended conformation of the β6/7 linker separates these two Trp residues by >10 Å (Figure 5D), we found that both are also absolutely essential for *Cac*Kam activity as substitution of either residue with Ala or Phe inactivates the enzyme in the kanamycin MIC assay (Table 1). The essential nature of R43/ R73 and W203/R206, thus necessitates a substantial, functional reorganization of the β6/7 linker. Specifically, we propose that initial docking of the β1/2 and β2/3 linker surface on the 30S, including interaction of R43 and R73 with the 16S rRNA, releases W203 and promotes β6/7 linker closure to reposition R206 and W203 to couple substrate binding with A1408 flipping and stabilization in the enzyme active site.

### Role of β6/7 linker reorganization in catalytic regulation

**Figure 6.**
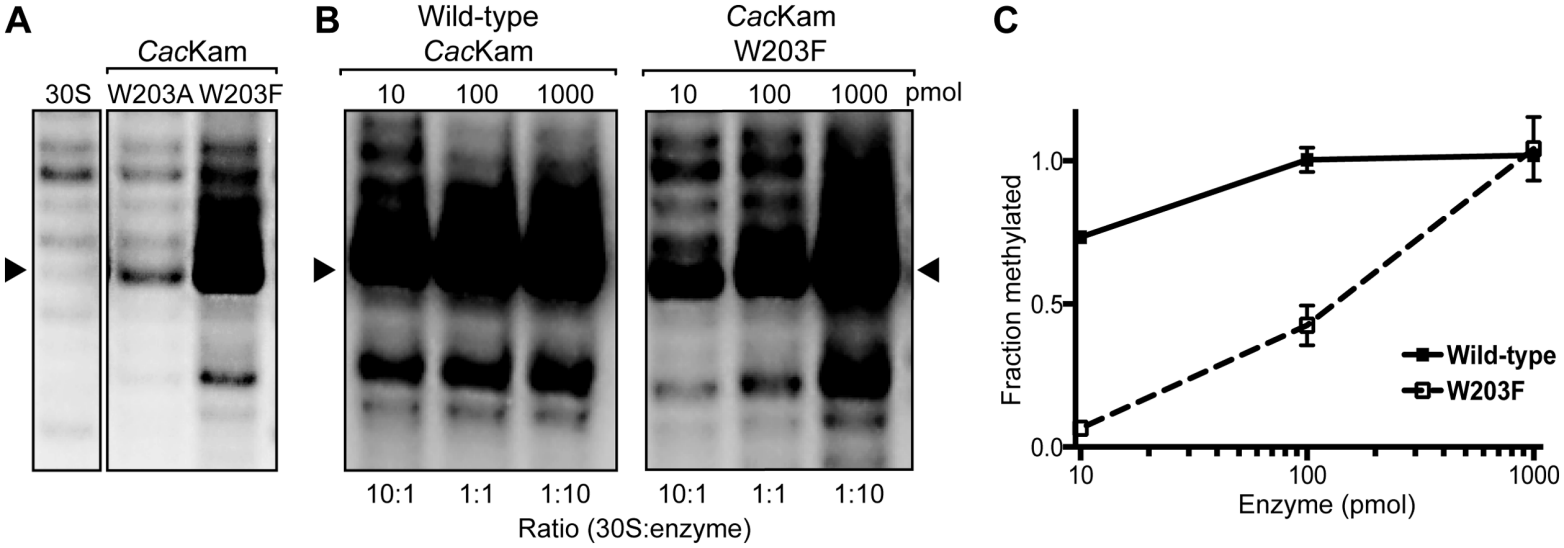
Primer extension analysis of m^1^A1408 modification by W203 substituted *Cac*Kam proteins. (**A**) Autoradiogram of RT primer extension of 16S rRNA from 30S subunits treated with W203A or W203F substituted *Cac*Kam protein reveals strong m^1^A1408 methylation for the latter enzyme only (indicated by strong termination at nucleotide C1409, indicated with an arrow). (**B**) Representative comparison of RT primer extension following wild-type *Cac*Kam and *Cac*Kam-W203F modification of 30S using an increasing enzyme to substrate ratio. (**C**) Quantification of RT analyses from panel B.

To determine the exact functional role of the *Cac*Kam β6/7 linker and W203 in particular, we directly compared the ability of wild-type, W203A- and W203F-substituted proteins to methylate A1408 using an *in vitro* methylation assay. Remarkably, despite our observation that neither protein with a substituted W203 could support bacterial growth in the presence of kanamycin (Table 1), *Cac*Kam-W203F displayed robust ability to catalyze m^1^A1408 modification under the conditions used in our standard assay (with 2-fold enzyme excess; Figure 6A). In contrast, *Cac*Kam-W203A showed minimal methylation capacity, consistent with its inactivity in the MIC assay. We next compared the catalytic efficiency of *Cac*Kam-W203F with the wild-type enzyme using different enzyme to substrate ratios (1:10, 1:1 and 10:1). Methylation by the wild-type enzyme was essentially the same under each condition as expected (Figure 6B and C). In contrast, W203F showed minimal activity at the lowest enzyme concentration, with increasing methylation correlated with increasing enzyme to 30S ratio (Figure 6B and C).

The retained *in vitro* methylation activity of *Cac*Kam-W203F is consistent with a requirement for an aromatic stacking to position the A1408 target base for methylation, as observed in the 30S–NpmA complex structure (18). This role of the conserved Trp residue can be fulfilled by a Phe but not an Ala side chain. However, why *Cac*Kam-W203F is unable to confer resistance to antibiotic in the MIC assay is not clear. The parsimonious explanation for this observation, as well as the concentration dependence of *in vitro* 30S methylation by *Cac*Kam-W203F, is that W203 plays an additional, critical role in enzymatic turnover that cannot be fulfilled by either Ala or Phe substitution. Specifically, we suggest that reversal of the β6/7 linker remodeling to reestablish interaction of W203 with R43 and R73 is necessary to complete the final step of product release from the enzyme. This idea is also supported by the similar sensitivity to proteolysis of the *Cac*Kam-W203F and W203A variants (Supplementary Figure S3) and *in silico* analysis of a W203F substitution using our wild-type *apo* protein structure which suggests the β6/7 linker would need to be further extended to maintain the cation–π stacking interaction with Phe. Additionally, we speculate that the relative stability of W203 interaction with R43/R73 in the free enzyme and with the A1408 target base in the 30S–enzyme complex may be tuned to facilitate this dynamic substrate-dependent remodeling. In this regard, it is noteworthy that the cation-π stacking interactions of W203 are close to but do not adopt the precise geometry for optimal stability (31,32). In summary, our observations suggest that *Cac*Kam W203 plays at least two critical roles in m^1^A1408 modification via target base positioning and regulation of enzymatic turnover, the latter of which specifically requires the presence of a Trp residue at this position.

The equivalent residue to *Cac*Kam W203 is conserved in all known 16S rRNA (m^1^A1408) methyltransferases. For NpmA, only a W197A substitution has been tested (14). While this variant both inactivates the enzyme activity *in vitro* and ablates its ability to confer resistance in bacteria, these observations are readily explained by this residue's established role in A1408 positioning in the enzyme active site. For KamB, both W193A and W193F render the enzyme unable to confer resistance in bacteria but these variants were not tested for activity *in vitro*. Thus, whether additional essential functional roles for this conserved Trp residue are employed by other family members requires further detailed investigation.

## CONCLUSIONS

Dynamic reorganization of flexible loops and other structural elements are critical for activity of diverse enzymes including protein kinases, fructose-1,6-bisphosphate aldolase, lipase, enolase and HIV protease (33–38). In several DNA methyltransferases, increased active site hydrophobicity resulting from loop closure is proposed to facilitate the methyl transfer reaction (39). Additionally, for the tRNA methyltransferase Trm5, ordering of a protein loop upon substrate binding was proposed to be important for stabilization of the flipped G37 base (40).

The present work has revealed a novel conformation of the *Cac*Kam β6/7 linker which harbors several residues essential for 16S rRNA recognition and target base positioning in other m^1^A1408 methyltransferases. Our results suggest that 30S substrate-dependent remodeling of this structure is necessary to position these functionally critical residues and this reorganization may additionally play a novel role in regulating *Cac*Kam enzymatic turnover via the interactions made by W203. However, determining the catalytically competent state of *Cac*Kam and the complete molecular details of this novel mechanism of enzyme regulation will require structural analyses of the 30S–enzyme complex. The sole current 30S–enzyme complex structure revealed that catalysis of m^1^A1408 modification by the aminoglycoside-resistance methyltransferases is likely to be largely dependent on the precise positioning of the target base and cosubstrate in close proximity. Whether the novel mechanism of controlling substrate specificity revealed in this work is unique to *Cac*Kam, and necessitated by a specific requirement for more stringent control of m^1^A1408 modification in *C. acidiphilia*, or more widely exploited by other 16S rRNA (m^1^A1408) methyltransferases is another important open question.

The function of the β6/7 linker as a dynamic regulator of enzyme activity has potentially broad significance for the catalytic reaction mechanism of the 16S rRNA (m^1^A1408) methyltransferases not apparent from their structural snapshots. In addition, our finding that substitutions of residues surrounding the bound SAM cosubstrate have starkly different effects on *Cac*Kam or KamB activity compared to equivalent changes in NpmA indicates that distinct mechanisms of action are present within the m^1^A1408 aminoglycoside-resistance methyltransferase family. Whether such variation also exists among the m^7^G1405 aminoglycoside-resistance methyltransferases which are currently more prevalent among human and animal pathogenic bacteria remains to be determined. However, understanding the molecular mechanisms and potential for variation among enzymes acquired by pathogenic bacteria will be essential for any efforts to develop specific inhibitors of these aminoglycoside-resistance determinants.

## ACCESSION NUMBERS

RCSB PDB: 4X1O, 5D1N, 5D1H, 5BW4, 5BW5.

## Supplementary Material

SUPPLEMENTARY DATA
