# Supplementary material for: Functional dichotomy in the 16S rRNA (m1A1408) methyltransferase family and control of catalytic activity via a novel tryptophan mediated loop reorganization
Source: Nucleic Acids Res. 2015 Nov 24;44(1):342–53. doi: 10.1093/nar/gkv1306 (PMC4705659; doi:10.1093/nar/gkv1306)
Supplement: SUPPLEMENTARY DATA [file supp_44_1_342__index.html]

Functional dichotomy in the 16S rRNA (m1A1408) methyltransferase family and control of catalytic activity via a novel tryptophan mediated loop reorganization — SUPPLEMENTARY DATA 

# Functional dichotomy in the 16S rRNA (m1A1408) methyltransferase family and control of catalytic activity via a novel tryptophan mediated loop reorganization

## SUPPLEMENTARY DATA

- SUPPLEMENTARY DATA
