# Supplementary material for: Functional dichotomy in the 16S rRNA (m1A1408) methyltransferase family and control of catalytic activity via a novel tryptophan mediated loop reorganization
Source: Nucleic Acids Res. 2015 Nov 24;44(1):342–53. doi: 10.1093/nar/gkv1306 (PMC4705659; doi:10.1093/nar/gkv1306)
Supplement: SUPPLEMENTARY DATA [file supp_gkv1306_nar-02997-h-2015-File008.pdf]

SUPPLEMENTARY DATA

Functional dichotomy in the 16S rRNA (m^{1}A1408) methyltransferase family and control of catalytic activity via a novel tryptophan mediated loop reorganization

Marta A. Witek and Graeme L. Conn*

Department of Biochemistry, Emory University School of Medicine, Atlanta GA 30322, USA.

*To whom correspondence should be addressed. Tel:+1 (404) 727-5965; Fax: (404) 727-2738 ; E-mail: gconn@emory.edu

SUPPLEMENTARY TABLES 1-2

Table S1. X-ray data collection and structure refinement statistics.

Table S2. Differential effects on enzyme activity of substitutions at conserved residues in the *Cac*Kam, KamB and NpmA SAM-binding pockets.

SUPPLEMENTARY FIGURES S1-S5

Figure S1. Interactions of W203 determine the β6/7 linker conformation.

Figure S2. Binding of either cosubstrate (SAM) or by-product (SAH) induces flexibility in the β6/7 linker.

Figure S3. Limited trypsin proteolysis of the β6/7 linker in wild-type and substituted *Cac*Kam proteins.

Figure S4. Comparison of the SAM-binding pockets of wild-type and W203 substituted *Cac*Kam.

Figure S5. Roles of β6/7 linker residues in activities of *Cac*Kam, KamB, Kmr and NpmA.

Table S1. X-ray data collection and structure refinement statistics.

	***Cac*Kam**	***Cac*Kam:SAH**	***Cac*Kam-W203A**	***Cac*Kam-W203A:SAM**	***Cac*Kam-D21A**
PDB code	4X1O	5D1N	5D1H	5BW4	5BW5
Space group	$P2_12_12_1$	$P2_12_12_1$	$P2_12_12_1$	$P2_12_12_1$	$P2_12_12_1$
Resolution (Å)[a]	50.00-1.70 (1.73-1.70)	50.00-2.70 (2.75-2.70)	50.00-2.80 (2.85-2.80)	50.00-2.10 (2.14-2.10)	50.00-2.50 (2.54-2.50)
Cell dimensions					
a, *b*, *c* (Å)	42.38, 66.11, 80.41	42.74, 65.97, 82.87	66.01, 84.11, 94.37	65.72, 83.23, 93.87	42.95, 66.66, 82.61
α, β, γ (º)	90,90,90	90,90,90	90,90,90	90,90,90	90,90,90
Wavelength (Å)	1.000	1.000	1.000	1.000	1.000
R_{merge}[b]	0.072 (0.867)	0.127 (0.778)	0.107 (0.588)	0.082 (0.856)	0.320 (0.615)
$I / \sigma I$	32.0 (2.2)	11.2 (1.9)	21.4 (3.8)	26.9 (2.1)	15.8 (2.4)
Completeness (%)	99.0 (98.4)	99.2 (100.0)	93.9 (94.9)	99.7 (99.9)	97.9 (86.0)
Redundancy	4.6 (4.7)	3.9 (4.0)	4.3 (4.2)	4.0 (4.0)	3.5 (2.2)
Total. reflections (used)	116,813 (24,665)	26,151 (6,302)	53,650 (12,210)	121,465 (27,026)	29,474 (8,023)
R_{work} / R_{free}[d]	0.177/0.221	0.213/0.281	0.183/0.234	0.183/0.229	0.239/0.292
Number of atoms	1,832	1,709	3,085	3,320	1,676
Protein	1,644	1,687	3,075	3,264	1,641
Water	188	22	10	56	35
B-factors					
Protein	30.70	34.20	54.30	50.20	50.90
Water	41.80	32.40	41.90	45.10	45.10
Ramachandran Plot					
Favorable (%)	98.62	95.91	96.27	96.45	96.77
Outliers (%)	0.00	0.00	0.50	1.42	0.00
R.m.s. deviations					
Bond lengths (Å)	0.007	0.002	0.003	0.022	0.003
Bond angles (º)	1.088	0.663	0.754	2.04	0.736

[a]Values in parenthesis are for the highest resolution shell.
[b]$R_{merge} = \Sigma hkl\ \Sigma i \,|\, Ii\ (hkl) - \langle I(hkl)\rangle \,|\, / \Sigma hkl\ \ \Sigma i\ Ii(hkl)$.
[c]Figure of merit (FoM), $m = \cos(\alpha - \alpha_{best})$.
[d]$R_{work} = \Sigma hkl \,|\, F_o\ (hkl) - F_c\ (hkl) \,|\, / \Sigma hkl \,|\, F_o\ (hkl)$, where F_o and F_c are observed and calculated structure factors, respectively. R_{free}, applies to the 5% of reflections chosen at random to constitute the test set.

TABLE S2. Differential effects on enzyme activity of substitutions at conserved residues in the *Cac*Kam, KamB and NpmA SAM-binding pockets.

Enzyme	Kanamycin MIC, μg/ml	Binding affinity, K_d (μM)	
		**SAM**	**SAH**
*Cac*Kam-E94A	16	29	2.2
KamB-E88A	256	56	5.3
NpmA-E88A[a]	1024	NB[b]	NB[b]
*Cac*Kam-S201A	16	110	1.6
KamB-T191A	16	48	5.9
NpmA-S195A[a]	1024	NB[b]	33

[a]Data are from reference (14).
[b]NB, denotes no binding detected.

SUPPLEMENTARY FIGURES

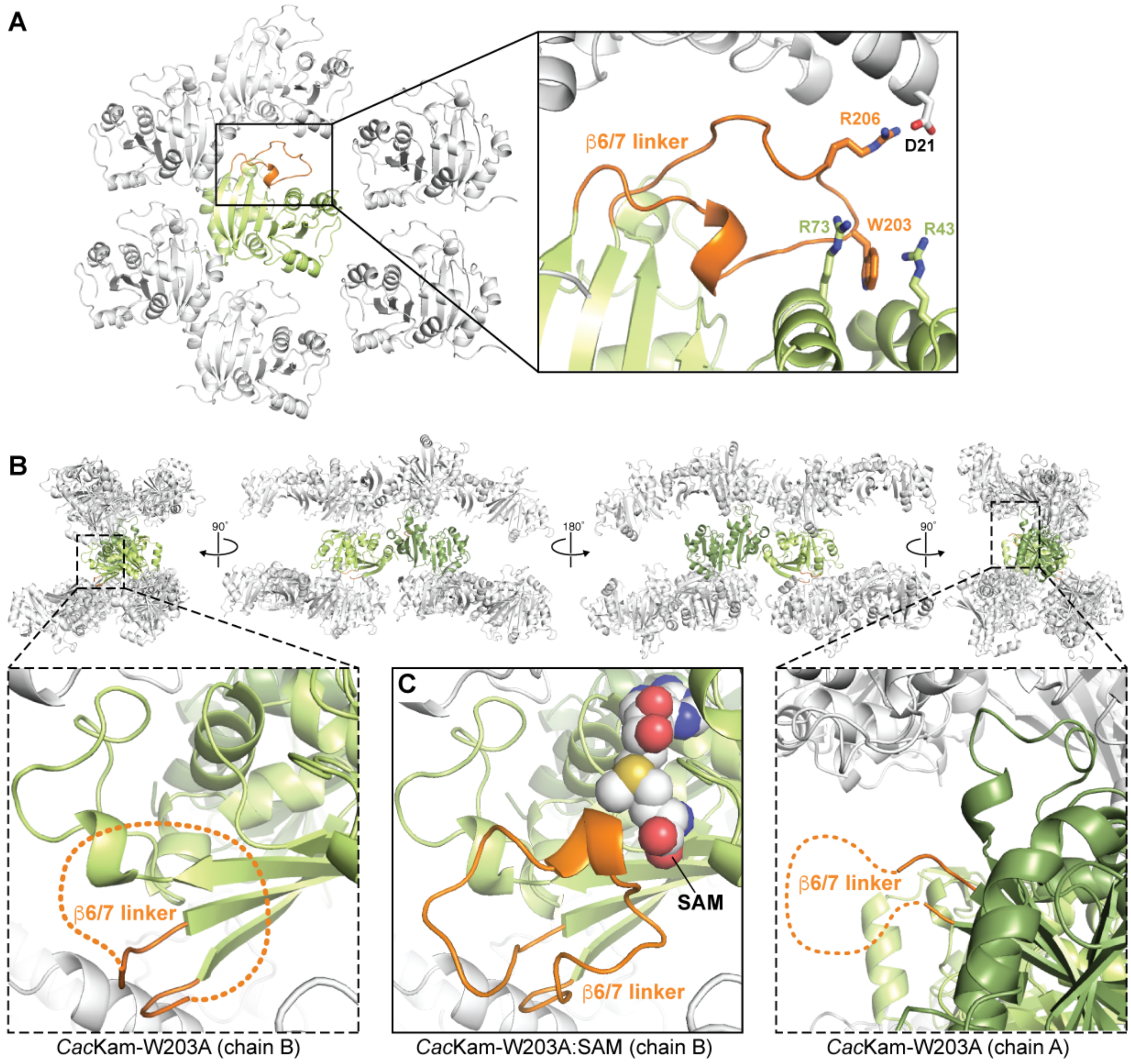

FIGURE S1. Interactions of W203 determine the β6/7 linker conformation. *A,* The *apo Cac*Kam structure with a single molecule in the asymmetric unit highlighting the two major interactions that stabilize the novel β6/7 linker conformation: an *inter*molecular salt bridge between R206 with D21, and an *intra*molecular interaction involving double cation-π stacking around W203 by two Arg residues, R43 and R73. ***B,*** W203A-substituted *Cac*Kam crystallizes with a new packing arrangement (two molecules in the asymmetric unit) in which the β6/7 linker is unrestrained by crystal packing, and for which no density was observed to allow modeling of the loop in either molecule. Zoomed views of the region surrounding the disordered β6/7 linker in chains A and B are shown boxed on the *right* and *left*, respectively. ***C,*** A zoomed view of the structure of the β6/7 linker in chain B of the *Cac*Kam-W203A:SAM complex (equivalent to the *left* zoomed view for *apo Cac*Kam-W203A). Unambiguous density allowed modeling of the loop in a 'closed' conformation that caps the SAM-binding pocket.

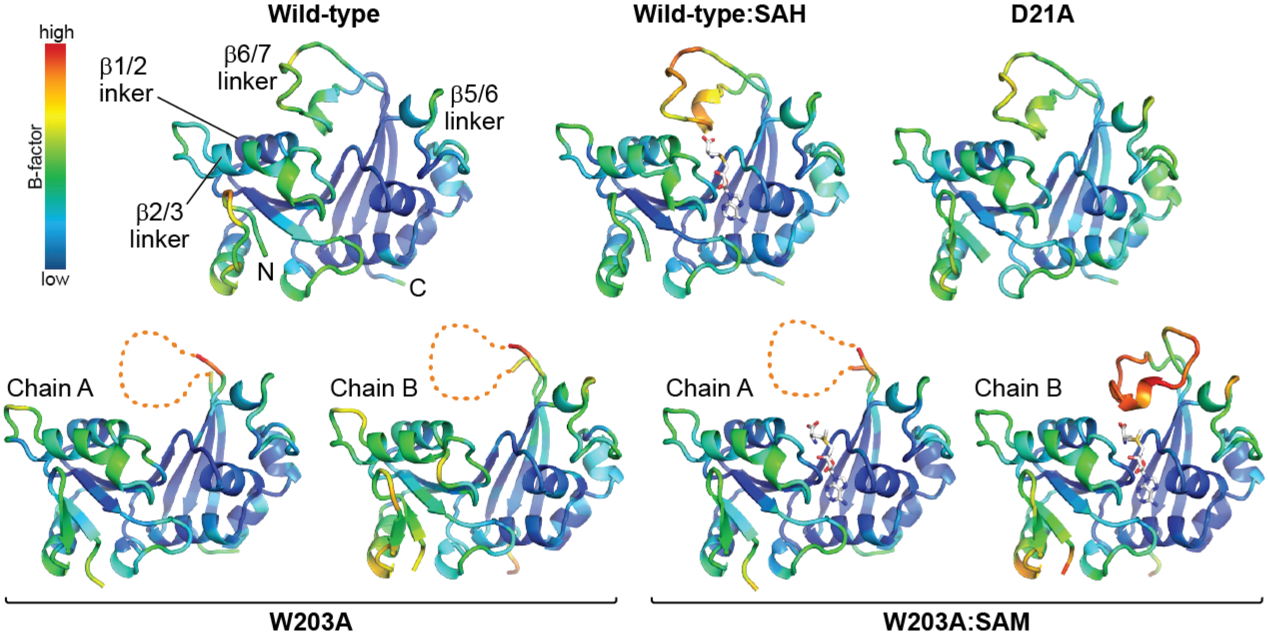

FIGURE S2. Binding of either cosubstrate (SAM) or by-product (SAH) induces flexibility in the β6/7 linker. Structures of wild-type and single amino acid substituted variants shown as cartoons colored by B-factor. Regions of lowest and highest B-factor are shown in blue and red, respectively (full scale shown *top left*).

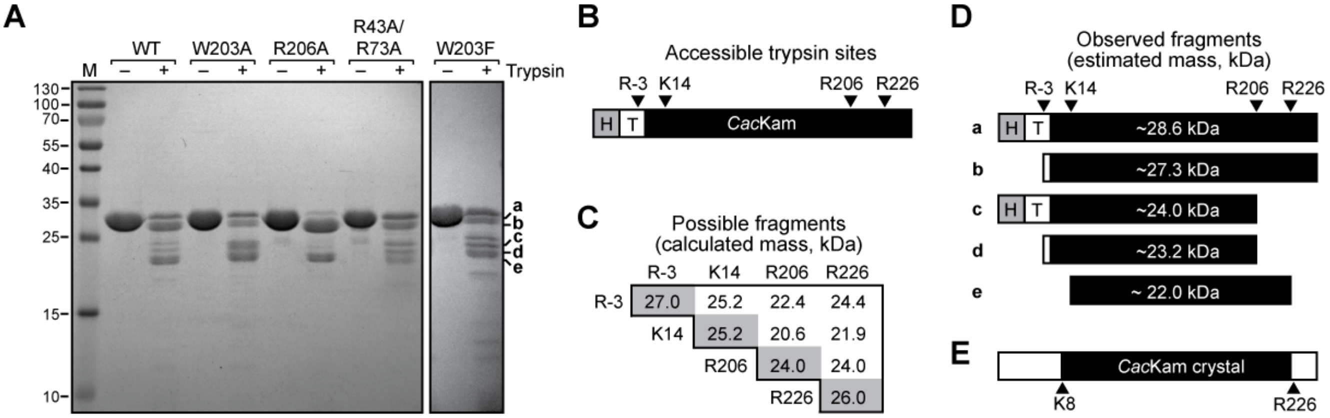

FIGURE S3. Limited trypsin proteolysis of the β6/7 linker in wild-type and substituted *Cac*Kam proteins. ***A***, 16% SDS-PAGE analysis of untreated (-) and trypsin treated (+) wild-type or variant *Cac*Kam protein. At least five distinct bands (a-e), including the full-length protein (band a), are observed for all except *Cac*Kam-R206A which has only three (a,d, and e). ***B***, Schematic of the full-length protein with predicted accessible trypsin cleavage sites and the locations of N-terminal 6×His tag (H) and thrombin protease recognition site (T) indicated. ***C***, Possible fragment masses generated by trypsin digest at the putative sites shown in *panel B*. ***D***, Additional schematics show the predicted fragments corresponding to each observed band (a-e) on the gel in *panel A*. ***E***, The schematic shows the region of the protein modeled (black background) in the *apo* wild-type *Cac*Kam structure.

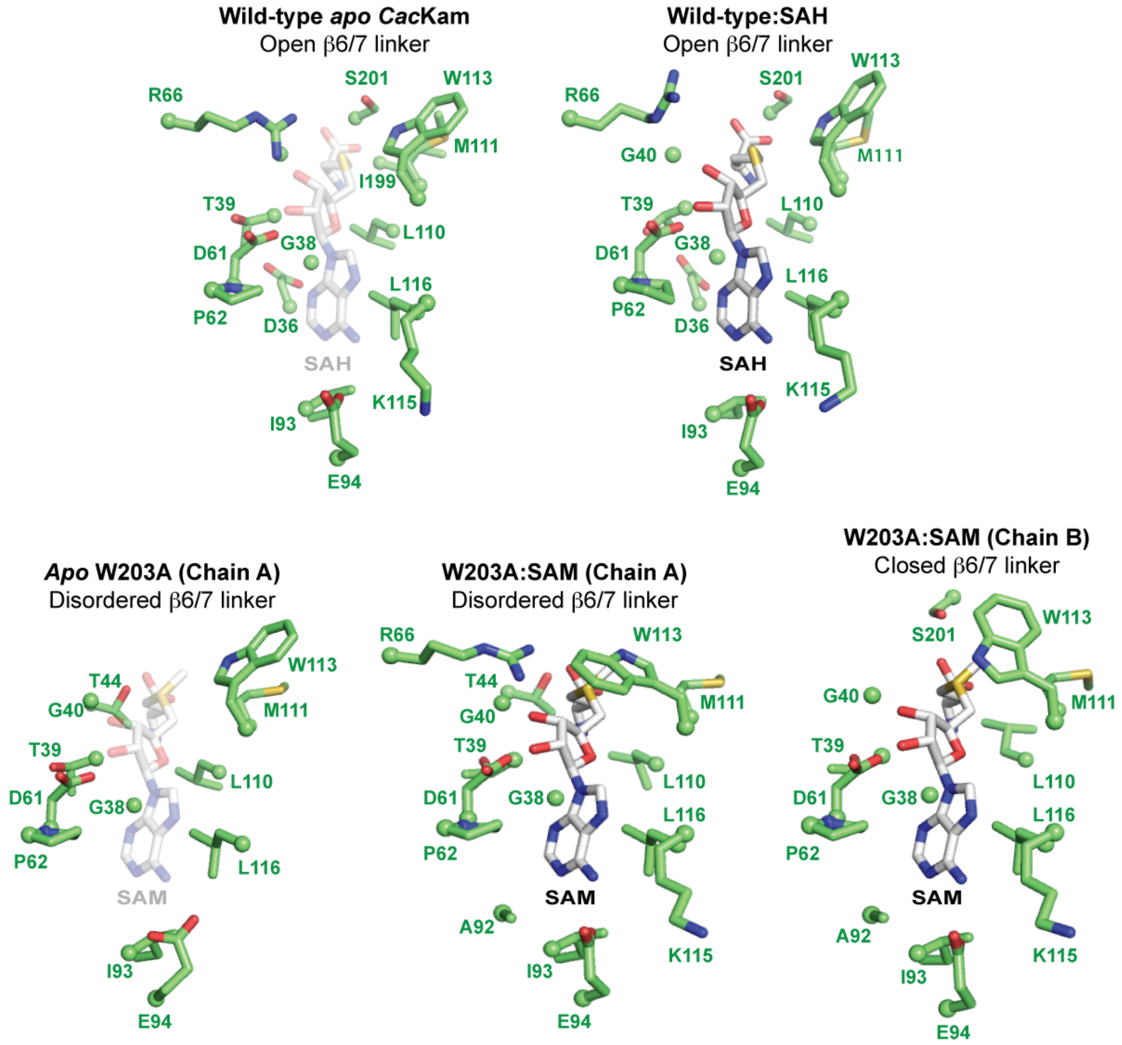

FIGURE S4. Comparison of the SAM-binding pockets of wild-type and W203 substituted *Cac*Kam. Residues forming the SAM-binding pockets in *apo* and ligand-bound wild-type *Cac*Kam (*top*) and *Cac*Kam-W203A (*bottom*). For *apo* protein structures (*left*) the position of SAM or SAH is indicated with the ligand (shown as semi-transparent sticks) taken from the equivalent complex structure (*right*). The SAM-binding pocket in each protein is formed by a largely conserved set of residues with only minor changes observed (e.g. adoption of different rotameric states by S201, R66 and W113 side chains).

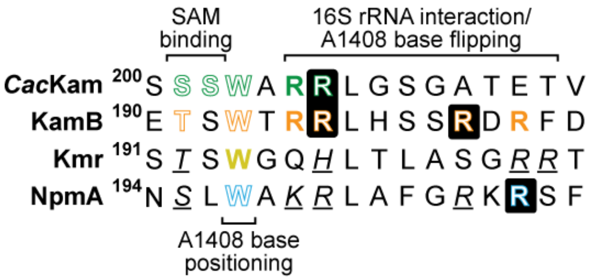

FIGURE S5. Roles of β6/7 linker residues in activities of *Cac*Kam, KamB, Kmr and NpmA. Highlighted residues indicate the effect of tested single amino acid substitutions where these inactivate enzymatic activity in kanamycin MIC assays (MIC ≤ 10-16 μg/ml; colored outline font), have intermediate effect on activity (MIC ~100-600 μg/ml; colored) and those with no or minimal impact (MIC ≥ 800 μg/ml; underlined italic font). Residues shown (NpmA) or proposed (KamB and *Cac*Kam) to be involved in base flipping are additionally denoted by a black background.
